# Gaps in knowledge regarding the diagnostic criteria and management of PCOS in Germany: An anonymous web-based survey

**DOI:** 10.1016/j.heliyon.2024.e40431

**Published:** 2024-11-19

**Authors:** Konstantin Hofmann, Melody Oehler, Christian Ruckes, Anna Dionysopoulou, Kathrin Stewen, Lina Judit Schiestl, Yaman Degirmenci, Susanne Theis, Christine Skala, Annette Hasenburg, Roxana Schwab

**Affiliations:** a Department of Obstetrics and Gynecology, University Medical Center of Johannes Gutenberg University Mainz, Germany; b University Medical Center of Johannes Gutenberg University Mainz, Germany; c Institute of Medical Biostatistics, Epidemiology, and Informatics, University Medical Center of Johannes Gutenberg University Mainz, Germany

**Keywords:** PCOS, Gynecologists, Gynecological endocrinology, Gaps in knowledge, Survey

## Abstract

**Introduction:**

Polycystic Ovary Syndrome (PCOS) is a multifactorial condition that can include a myriad of symptom complexes. A study in North America demonstrated that a significant percentage of physicians were unaware of crucial aspects of PCOS. This study aimed to examine the level of knowledge about PCOS among physicians in Germany.

**Methods:**

An anonymous cross-sectional online questionnaire was distributed to all gynecological clinics and fertility centers in Germany. The responsible gynecologists in service were contacted and they were asked to distribute the questionnaire among the employed physicians in their clinic.

**Results and discussion:**

The questionnaire was completed 206 times. 92 (65.7 %) of all respondents were board-certified gynecologists without specialty training in reproductive medicine and gynecologic endocrinology (Non-RMGE), 48 (34.3 %) had completed this training (RMGE). RMGE were more likely to know the correct criteria for the diagnosis of PCOS (97.9 % vs. 51.3 %; p < 0.001) and were able to name a higher number of correct symptoms that may be associated with PCOS (20.8 vs. 14.0; p < 0.001) than Non-RMGE (B = 4.530, CI 1.379–7.680; p = 0.006). The preferred general treatments and fertility treatments for PCOS patients also differed significantly between the two groups (p = 0.002, p < 0.001). The participants were asked how doctors and healthcare professionals could be best supported to improve the care of PCOS patients. Over 58.3 % of participants considered the creation of a PCOS website dedicated to healthcare professionals to be valuable.

**Conclusion:**

This study is the first to identify knowledge gaps about PCOS among physicians in Germany. The findings also highlight the potential disparities in PCOS knowledge between hospital and fertility center settings, emphasizing the need for improved training to ensure consistent and high-quality care for PCOS patients. The participants preferred a dedicated website for health professionals, indicating a demand for easily accessible information and training resources.

## 1 Introduction

Polycystic Ovary Syndrome (PCOS) is the most common endocrine disorder of women of reproductive age worldwide [[1], [2], [3]]. The Global Burden of Disease Study of the Bill and Melinda Gates Foundation estimates the age-standardized point prevalence globally at 1667.8 per 1,000,000 women (Germany: 2924.8 per 100,000 women) - with an increasing trend. Since 1990, the prevalence of the condition has increased globally by 30.4 % (Germany: 26.3 %) [4].

The diagnostic criteria for PCOS are regulated in the joint PCOS guideline of 2023. These are based on the Rotterdam criteria and consist of three parts: PCO morphology of the ovary or elevated anti-Müllerian hormone (AMH), hyperandrogenemia or clinical androgenization, and irregular cycles. Two of these three criteria must be fulfilled to make the diagnosis of PCOS [1]. In addition, there are other diagnostic systems, such as those of the National Institute of Health (NIH) and the Androgen Excess and PCOS Society, which are not recommended by the European Society of Human Reproduction and Embryology (ESHRE) [1,5].

PCOS is a multifactorial condition that can include symptom complexes such as excess weight, insulin resistance, cardiovascular disorders, depression, anxiety, and various other disorders [6,7]. These phenomena can occur not only in premenopausal patients but also play an important role in postmenopausal women. Still, they are often not attributed as accompanying factors of PCOS. Thus, even in postmenopausal patients, one observes a weight-independent increased risk for Type II diabetes mellitus (T2DM) or mental illnesses such as depression and anxiety disorders [8]. Preventive measures and tailored therapy can significantly influence the outcome of PCOS [1].

PCOS plays an important role in the care of patients with fertility problems. While women with PCOS can become pregnant, they often require fertility treatment [9]. The accompanying factors of PCOS significantly influence the success of fertility treatment. PCOS patients with higher weight or cardiovascular diseases have a significantly lower probability of conceiving [1].

However, not only do the manifestations of PCOS play a role in the conception of pregnancy itself, but also, they are relevant for the course of the pregnancy. For example, PCOS patients have a higher risk of gestational diabetes, hypertensive pregnancy disorders, and premature birth [10,11]. Children of PCOS patients have an increased risk of developing PCOS, obesity, or T2DM [12].

Therefore, early diagnosis of PCOS is important for the patients as well as their relatives. Studies showed an increased risk for parents and siblings of PCOS patients to suffer from T2DM, metabolic syndrome, and dyslipidemia. This applies not only to female family members but also to male ones [13]. Therefore, metabolic assessment of first-degree relatives is also recommended after the diagnosis of PCOS [1].

However, there is a chance to avoid at least some of these undesirable events for PCOS patients. Early detection of PCOS can allow for timely interventions or recommendations to improve the outcome of PCOS patients in general and for pregnancy in particular [14]. The first-line therapy for PCOS consists of lifestyle modification (increased physical activity, healthy diet), and for obese patients, weight reduction. For example, a recent study by Rezaei et al. showed that aerobic exercise improves the hormonal profile, overall health, and quality of life of PCOS patients [15,16].

In addition, there is a variety of other treatment options for PCOS that must be tailored to the individual needs of the patient. If the patient does not have a desire for pregnancy, the therapy is focused on reducing the individually experienced symptom burden [1].

Many PCOS patients suffer from manifestations of hyperandrogenism, such as acne, hair loss, or increased body hair. Conservative measures such as hair removal via shaving or laser therapy can improve hirsutism, while oral contraceptives can reduce hyperandrogenism and thereby decrease the symptom load [17,18]. Hormonal preparations also provide protection for the endometrium, thus lowering the risk of PCOS patients developing endometrial carcinoma [19].

Fertility treatments for PCOS patients also offer various treatment options. For instance, in cases of metabolic stress on patients, in addition to the aforementioned lifestyle modifications, the administration of metformin can lead to the normalization of the menstrual cycle and promote pregnancy. Additionally, ovulation inducers or Selective Estrogen Receptor Modulators (SERMs) such as clomiphene are commonly used [1].

This makes the diagnosis and treatment of PCOS complex and challenging for the treating physicians. A study involving 1385 participants PCOS patients revealed that one-third of the women took more than two years to receive the correct diagnosis. It was also necessary to consult multiple healthcare professionals. Additionally, they reported dissatisfaction with the information provided about their condition during the diagnostic process. There was a correlation between satisfaction with the information provided about PCOS and satisfaction with the diagnosis. The longer the time until the correct diagnosis was made, and the more healthcare professionals needed to be consulted, the greater the dissatisfaction with the PCOS diagnosis [20].

At the same time, knowledge about PCOS within the field of gynecology and even among reproductive medicine specialists and gynecological endocrinologists appears to be unevenly distributed. A study by Dokras et al. involving 630 practicing physicians in the United States demonstrated that a significant percentage of the respondents were unaware of crucial aspects of PCOS and the currently recommended criteria for the diagnosis and therapy of PCOS [21].

The aim of studying gynecologists' knowledge of criteria for PCOS diagnosis and treatment was to assess and enhance their proficiency in recognizing and managing PCOS effectively. This includes evaluating their knowledge of the diagnostic criteria and adherence to recommended treatment strategies. Additionally, the study sought to identify gaps or misconceptions in gynecologists' understanding of PCOS, with the objective of improving patient care outcomes through targeted education and training initiatives.

## 2 Methods

An anonymous cross-sectional online study was initiated by the Clinic for Obstetrics and Women's health of the University Medical Center Mainz. Data collection was carried out through an online questionnaire using the SoSciSurvey software. SoSciSurvey is an online platform that is frequently used in the academic context in the German-speaking region [22]. The survey was conducted from December 4, 2023, to January 17, 2024.

The invitation to the survey was digitally distributed to all gynecological clinics in Germany. To achieve this, the responsible gynecologists in service at all clinics in Germany were contacted, and they were asked to distribute the questionnaire among the employed physicians in their clinic. Additionally, fertility centers listed in the German IVF-Register (DIR) were contacted, and the gynecologists working there were invited to participate. Three reminders were sent in December 2023 and January 2024.

### 2.1 Inclusion and exclusion criteria

All physicians in the field of gynecology nationwide were eligible to participate. Participation in the study had to be voluntary. The inclusion criteria were listed on the online portal directly after the participant's briefing in the anonymous questionnaire, and participants had to actively click on them. Furthermore, physicians who did not consent to the survey were excluded. All study participants agreed to participate in the study and to the publication of the results.

### 2.2 Parameter

For the purpose of international comparability, the questionnaire from the aforementioned North American study by Dokras et al. was translated by ChatGPT 3.5 (OpenAI; USA) into German [21]. The questionnaire was checked for errors by three German native speakers and adapted for the German healthcare system.

Sociodemographic variables were collected, such as age, professional experience, membership in professional societies, and the status of any specialized training in the field of reproductive medicine and gynecological endocrinology. The level of training of the participants was distinguished into board-certified physicians (BC), non-board-certified physicians (NON-BC), BC with specialist training in reproductive medicine and gynecological endocrinology (RMGE) and BC without a specialist training in reproductive medicine and gynecological endocrinology (Non-RMGE). The survey also aimed to gather information on participants' comprehension and personal evaluation of PCOS. The complete questionnaire can be accessed in the appendix.

### 2.3 Statistical analysis

We anticipated around 150 individuals (about 20 % of the requested individuals) to participate in this descriptive survey. Power analyses were performed utilizing PROC POWER in SAS V.9.4 to estimate confidence intervals (with a power exceeding 99.9 %, proportions (e.g., 0.65 for Non-RMGE and 0.80 for RMGE) ranging from 0.65 to 0.80, and a half-width confidence interval of 0.08).

The data were analyzed using explorative data analysis and descriptive statistics. For this purpose, we used „IBM SPSS Statistics Version 29“. Means, standard deviations, frequency, and percentages were used to describe the characteristics of the participants.

All results of the χ2 test were provided. Means were compared using the *t*-test. P values describe the difference between the RMGE and Non-RMGE groups.

Variables with p-values less than 0.25 in the univariate regression model were then entered into the final model multivariate logistic regression by backward selection to determine the independence of the variables mentioned above for predicting the number of correctly named symptoms connected to PCOS [[23], [24], [25]]. The odds ratio (OR), regression coefficient B, standardized regression coefficient β, p-value, and 95 % confidence interval (95 % CI) were utilized to express the data.

### 2.4 Ethics declaration

According to German legal statutes, the committee of Rhineland Palatinate exempted the research protocol from ethical review and regulations, which stipulate that studies involving anonymous data collection do not require formal ethical approval (2023–17215). The study center and SoSciSurvey adhere to German and European legislation regarding data protection. The study was conducted according to the requirements of the "Declaration of Helsinki" [26].

## 3 Results

The survey invitation was distributed to a cohort of 747 gynecological clinics and fertility centers across Germany. Subsequently, the questionnaire obtained 319 clicks, with 242 individuals participating. 206 participants completed the questionnaire. The following dataset included the outcomes derived from the 206 questionnaires that were fully completed.

### 3.1 Demographic characteristics

The largest group of study participants was in the age range of 26–35 years (34.0 %). Among the participating BCs in gynecology and obstetrics (140, 68.0 %), 48 (34.3 %) had completed specialist training in reproductive medicine and gynecological endocrinology (RMGE), while 92 (65.7 %) had not (Non-RMGE). Table 1 shows the demographics of the study group. Additional data can be found in the appendix.

**Table 1:** Demographic of the respondents.

	Overall		Specialist training in reproductive medicine and gynecological endocrinology				
			RMGE		NON-RMGE		p
	n = 206		n = 48	34.3 %	n = 92	65.7 %	
Age (years)							0.491
**18**–**25**	5	2.4 %	0	0.0 %	0	0.0 %	
**26**–**35**	70	34.0 %	2	4.2 %	11	12.0 %	
**36**–**45**	48	23.3 %	15	31.3 %	30	32.6 %	
**46**–**55**	51	24.8 %	19	39.6 %	31	33.7 %	
**>56**	32	15.5 %	12	25.0 %	20	21.7 %	
Sex							0.443
**Female**	155	75.2 %	36	75.0 %	63	68.5 %	
**Male**	51	24.8 %	12	25.0 %	29	31.5 %	
Workplace							<0.001
**Fertility center**	55	26.7 %	35	72.9 %	16	17.4 %	
**Hospital**	151	73.3 %	13	27.1 %	76	82.6 %	
Type of hospital							0.029
**Primary care hospital**	37	24.5 %	0	0.0 %	18	23.7 %	
**Standard care hospital**	37	24.5 %	2	15.4 %	23	30.3 %	
**Maximum care hospital**	77	51.0 %	11	84.6 %	35	46.1 %	
Is there an outpatients‘ clinic for reproductive medicine and/or gynecological endocrinology in your hospital?							<0.001
**Yes**	42	20.4 %	10	76.9 %	16	21.1 %	
**No**	109	52.9 %	3	23.1 %	60	78.9 %	
Number of PCOS-patients seen annually							<0.001
**<50**	85	68.5 %	18	37.5 %	53	89.8 %	
**50**–**200**	37	29.8 %	30	62.5 %	4	6.8 %	
**>200**	1	0.8 %	0	0.0 %	1	1.7 %	
**None**	1	0.8 %	0	0.0 %	1	1.7 %	
Years since board certification (years							0.295
**0**–**5**	24	17.1 %	5	10.4 %	19	20.7 %	
**5**–**10**	32	22.9 %	11	22.9 %	21	22.8 %	
**11**–**15**	21	15.0 %	8	16.7 %	13	14.1 %	
**16**–**20**	19	13.6 %	9	18.8 %	10	10.9 %	
**21**–**25**	12	8.6 %	7	14.6 %	5	5.4 %	
**26**–**30**	19	3.6 %	6	12.5 %	13	14.1 %	
**31**–**35**	8	5.7 %	1	2.1 %	7	7.6 %	
**36**–**40**	5	3.6 %	1	2.1 %	4	4.3 %	

RMGE: completed specialist training in reproductive medicine and gynecological endocrinology; Non-RMGE: no completed specialist training in reproductive medicine and gynecological endocrinology, PCOS: Polycystic Ovary Syndrome; SD: standard deviation.

### 3.2 Diagnosis and clinical features of PCOS patients

Among the RMGE, all physicians report having previously diagnosed PCOS (Table 2). Participants were asked in the questionnaire to specify their criteria for diagnosing PCOS. The options included Rotterdam/ESHRE criteria, the definition of the Androgen Excess and PCOS Society, the criteria of the National Institutes of Medicine (NIH), "I don't know," and others. Under "others," participants provided free-text responses such as "Sonography and clinical presentation" and "suspected diagnosis," both of which were considered incorrect. Only participants who indicated in a previous question that they had already made the diagnosis of PCOS were allowed to answer this question. Almost all RMGE applied the ESHRE criteria for the diagnosis of PCOS, while 41 % of Non-RMGE claimed not to know which criteria were used for the diagnosis of PCOS (p < 0.001) (Table 2).

**Table 2:** Answers related to diagnosis and clinical features of PCOS patients.

	Overall		Specialist training in reproductive medicine and gynecological endocrinology				
			RMGE		NON-RMGE		
	n = 206		48	34.3 %	92	65.7 %	p
Have you ever diagnosed PCOS?							0.002
**Yes**	147	71.4 %	48	100.0 %	78	84.8 %	
**No**	59	28.6 %	0	0.0 %	14	15.2 %	
Knowing the diagnostic criteria							<0.001
**Yes**	103	70.1 %	47	97.9 %	40	51.3 %	
**I don't know**	36	24.5 %	0	0.0 %	32	41.0 %	
**No**	8	5.4 %	1	2.1 %	6	7.7 %	
Prevalence correctly estimated							0.005
**Yes**	96	46.6 %	30	62.5 %	43	46.7 %	
**No**	67	32.5 %	17	35.4 %	30	32.6 %	
**I don't know**	43	20.9 %	1	2.1 %	19	20.7 %	
Are PCOS-patients correctly diagnosed?							<0.001
**Correctly diagnosed**	39	18.9 %	17	35.4 %	10	10.9 %	
**Under-diagnosed**	84	40.8 %	24	50.0 %	42	45.7 %	
**Over-diagnosed**	20	9.7 %	4	8.3 %	11	12.0 %	
**I don't know**	63	30.6 %	3	6.3 %	29	31.5 %	
Most common reason for clinic attendance?							<0.001
**Hirsutism**	5	4.0 %	1	2.1 %	4	6.8 %	
**Infertility**	67	54.0 %	41	85.4 %	23	39.0 %	
**Menstrual disturbances**	49	39.5 %	6	12.5 %	30	50.8 %	
**Metabolic disorders**	3	2.4 %	0	0.0 %	2	3.4 %	
**Obesity**	0	0.0 %	0	0.0 %	0	0.0 %	
**Other**	0	0.0 %	0	0.0 %	0	0.0 %	
Do you ask your patients about family history of diabetes, cardiovascular disease, and/or PCOS?							0.076
**Yes**	69	55.6 %	33	68.8 %	30	50.8 %	
**No**	55	44.4 %	15	31.3 %	29	49.2 %	
Number of correct named symptoms connected to PCOS							<0.001
**Mean**	14.5		20.8		14.0		
**Median**	15		22		14		
**SD**	6.450		2.973		5.389		
**Minimum**	1		12		2		
**Maximum**	24		24		24		

RMGE: completed specialist training in reproductive medicine and gynecological endocrinology; Non-RMGE: no completed specialist training in reproductive medicine and gynecological endocrinology; PCOS: Polycystic Ovary Syndrome; SD: standard deviation.

The participants were asked whether they could associate specific symptoms with PCOS. Out of the listed symptoms, 24 were related to PCOS (e.g., loss of scalp hair or reduced quality of life), while 5 (e.g., ovarian cysts) were not. On average, 14.5 correct answers were given, with RMGE showing a significantly higher rate of correct answers than Non-RMGE (20.8 vs. 14.0; p < 0.001).

The distribution of responses from all study participants regarding symptoms associated with PCOS is listed in the appendix.

### 3.3 Management of PCOS patients

The options for PCOS therapies were based on the aforementioned study by Dokras et al. to establish comparability. However, in the context of fertility treatment for PCOS patients, letrozole has largely displaced clomiphene. The emergence of GLP-1 receptor agonists was also not taken into account. Other therapy options beyond those mentioned below were not indicated in the questionnaire.

Regarding the general treatment of PCOS patients without a current desire for children, RMGE indicated lifestyle modification (35.0 %) and oral contraceptives (26.0 %) as the most common therapies. Non-RMGE reported 27.0 % for lifestyle modification and 32.0 % for oral contraceptives (p = 0.002). In terms of fertility treatments, 26.1 % of RMGE prioritized clomiphene as the most common therapy over lifestyle modification at 24.6 %. The largest group of Non-RMGE (35.3 %) did not perform any fertility treatments. This is likely attributed to the workplace of Non-RMGE (Table 3).

**Table 3:** Answers related to the management of PCOS patients.

	Overall		Specialist training in reproductive medicine and gynecological endocrinology				
			RMGE		NON-RMGE		
	n = 206		48	34.3 %	92	65.7 %	p
General treatment most commonly prescribed							0.002
**Anti-androgens**	34	11.9 %	14	11.4 %	16	13.1 %	
**Laser depilation**	16	5.6 %	7	5.7 %	6	4.9 %	
**Lifestyle modifications**	87	30.5 %	43	35.0 %	33	27.0 %	
**Metformin**	51	17.9 %	23	18.7 %	22	18.0 %	
**Oral contraceptives**	85	29.8 %	32	26.0 %	39	32.0 %	
**Other**	6	2.1 %	4	3.3 %	1	0.8 %	
**None**	6	2.1 %	0	0.0 %	5	4.1 %	
Fertility treatment most commonly prescribed							<0.001
**Clomiphene**	52	20.3 %	36	26.1 %	10	11.8 %	
**Clomiphene + metformin**	28	10.9 %	20	14.5 %	5	5.9 %	
**Lifestyle modifications**	66	25.8 %	34	24.6 %	24	28.2 %	
**Metformin**	34	13.3 %	19	13.8 %	11	12.9 %	
**Ovulation inductors**	38	14.8 %	28	20.3 %	5	5.9 %	
**None**	38	14.8 %	1	0.7 %	30	35.3 %	
Most important long-term concern							0.019
**Infertility**	49	23.8 %	2	4.2 %	22	23.9 %	
**Cardiovascular diseases**	25	12.1 %	7	14.6 %	14	15.2 %	
**Obesity/T2DM**	84	40.8 %	29	60.4 %	39	42.4 %	
**Endometrial cancer**	33	16.0 %	7	14.6 %	13	14.1 %	
**Psychosocial problems**	11	5.3 %	3	6.3 %	2	2.2 %	
**Other**	4	1.9 %	0	0.0 %	2	2.2 %	

RMGE: completed specialist training in reproductive medicine and gynecological endocrinology; Non-RMGE: no completed specialist training in reproductive medicine and gynecological endocrinology; PCOS: Polycystic Ovary Syndrome; SD: standard deviation; T2DM: Type II diabetes mellitus.

### 3.4 Univariate and multivariate analysis

To investigate the relationship between the number of correctly named symptoms associated with PCOS and RMGE training, a univariate and multivariate analysis using linear regression was conducted. This analysis reveals a significant association for RMGE (univariate: p < 0.001) (Table 4).

**Table 4:** Univariate Analysis of factors possibly influencing the number of correctly named symptoms connected to PCOS.

	B	SD	β	p	95%-CI	
Age	2.191	0.368	0.385	<0.001	1.465	2.916
Male sex	0.124	1.044	0.008	0.905	−1.934	2.182
Working in a primary care hospital	−4.477	1.150	−0.304	<0.001	−6.749	−2.205
Working in a standard-care hospital	1.287	1.202	0.087	0.286	−1.089	3.662
Working in a maximum-care hospital	2.362	1.020	0.186	0.022	0.346	4.378
Never diagnosed PCOS	−7.541	0.845	−0.530	<0.001	−9.208	−5.875
Completed board certification (BC)	5.557	0.884	0.403	<0.001	3.815	7.299
No outpatients‘ clinic for reproductive medicine and gynecological endocrinology	−3.375	1.125	−0.239	0.003	−5.599	−1.152
RMGE	6.845	0.838	0.571	<0.001	5.188	8.502
Years since board certification	0.140	0.238	0.050	0.558	−0.331	0.612
Number of PCOS patients seen annually	3.055	0.776	0.336	<0.001	1.520	4.591

B: regression coefficient; β: standardized regression coefficient; CI: confidence interval; Non-RMGE: no completed specialist training in reproductive medicine and gynecological endocrinology; PCOS: Polycystic Ovary Syndrome; SD: standard deviation.

The multivariate regression model for the prediction of the number of correctly named symptoms connected to PCOS included the following possible predictors: age, years since board certification, working in a primary care hospital, working in a maximum care hospital, never diagnosed PCOS, no outpatients’ clinic for RMGE, number of PCOS patients seen annually and RMGE. Only RMGE (B = 4.530, CI 1.379–7.680; p = 0.006) was an independent factor for a higher number of correctly named symptoms connected to PCOS. (R^2^ = 0.131, corr. R^2^ = 0.115, SD = 4.980)

### 3.5 Support for clinicians and healthcare professionals

**Fig. 1:**
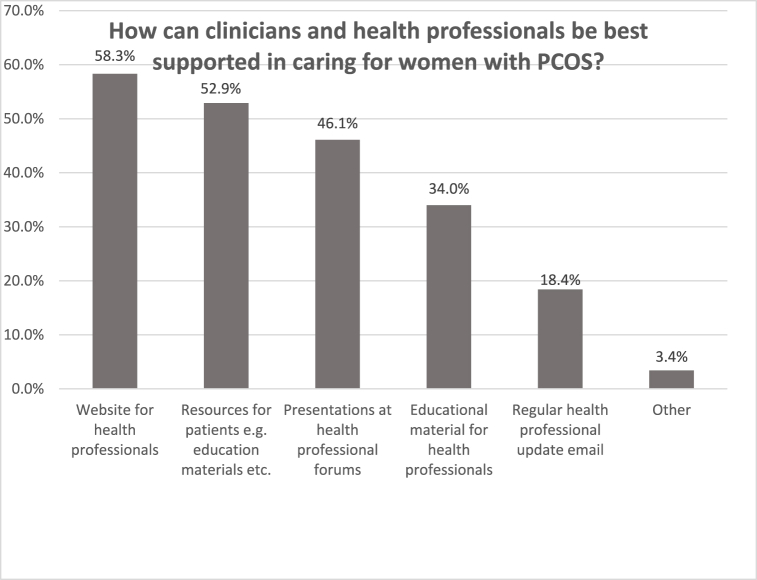
How can clinicians and health professionals be best supported in caring for women with PCOS? The participants were able to select as many answer options as they wished. The percentage of given selected answers of all participants is shown. PCOS: Polycystic Ovary Syndrome.

At the end of the questionnaire, participants were asked how doctors and healthcare professionals could be best supported to improve the care of PCOS patients. Over 58.3 % of participants considered the creation of a PCOS website dedicated to healthcare professionals to be valuable. Subsequently, it was mentioned that the generous provision of informational materials, such as brochures for patients, could be helpful (Fig. 1).

## 4 Discussion

This is the first study identifying knowledge gaps regarding PCOS among physicians practicing in Germany. Similar to the study by Dokras et al. from 2017, it must be noted that only 70.1 % of all respondents and only 51.3 % of BC were familiar with the recommended criteria for diagnosing PCOS. A significant portion of the respondents (24.5 % and 41.0 %) directly admitted, not knowing which criteria to apply. Interestingly, Non-BC performed better in this regard than BC.

97.9 % of RMGE used the recommended ESHRE criteria for diagnosing PCOS. This aligns with the results in North America, where 94.1 % of Reproductive Endocrinologists knew the criteria, while only 62.9 % of the study participants were familiar with them [21]. This is also in line with further studies on this topic that have identified differences in the knowledge and treatment of PCOS. Consistently, it is evident that RMGE or individuals in training for RMGE have greater knowledge of PCOS, and this significantly influences decision-making regarding PCOS [[27], [28], [29], [30]].

As Non-BC in training for gynecology and obstetrics naturally possess less experience in the diagnosis and treatment, potentially distorting the results, the study focused on RMGE and Non-RMGE. In Germany, only BCs can have completed specialized training in reproductive medicine and gynecological endocrinology. This lack of experience also explains why so many study participants have never diagnosed PCOS.

If PCOS patients present themselves in German hospitals (as there are also outpatient clinics and consultations where PCOS patients are treated by Non-RMGE), the chances are high that they will be treated differently compared to their RMGE colleagues. The ESHRE guideline published in 2023 recommends lifestyle modifications, including regular physical activity and a balanced diet, as the first-line non-fertility-related therapy. If this does not lead to an improvement in PCOS symptoms, oral contraceptives, dietary supplements such as myo-inositol, or metformin therapy in cases of insulin resistance or obesity are recommended [1].While RMGE preferred lifestyle modifications as a general PCOS therapy, Non-RMGE were more inclined to prescribe oral contraceptives. With regard to fertility treatments for PCOS patients, the guideline recommends an individualized approach. Although many PCOS patients can benefit from lifestyle modification for their fertility, pharmacological therapy for irregular cycles involves administering ovulation inducers [1,31]. In fertility treatments, RMGE were most likely to prescribe clomiphene from the given options, while Non-RMGE recommend lifestyle modifications. The willingness of RMGE to use pharmacological fertility therapies likely stems from their greater experience in this area. It is important to note, however, that letrozole has now replaced clomiphene as the first-line treatment for PCOS [1]. Additionally, most Non-RMGE did not perform fertility treatments at all. This may be due to a general shift of fertility treatments from hospitals to outpatient fertility centers in Germany in recent decades.

Overall, one could state that, following the results of this study, knowledge about PCOS among physicians in hospitals may be lower compared to fertility centers. This could possibly be attributed to the fact that fertility centers handle a higher number of PCOS patients, necessitating a more in-depth engagement with diagnostic criteria and therapeutic approaches. Knowledge levels' disparity may indicate a need for enhanced training and educational programs specifically targeting hospital doctors to ensure improved care and diagnostic capabilities for PCOS patients.

Furthermore, recent research could transform the clinical management of PCOS. Recent discoveries regarding the kisspeptin system and its role in regulating the hypothalamic–pituitary–gonadal axis indicate that it may be crucial for elucidating the shared yet contrasting mechanisms in both PCOS and endometriosis. Additionally, kisspeptin's involvement in metabolic, inflammatory, and pain pathways underscores its potential as a therapeutic target, enhancing our understanding of these conditions as interconnected yet opposing facets of female reproductive health [32].

In recent years, addressing the most apparent issues faced by PCOS patients, such as fertility treatment or androgenic symptoms, attention has shifted towards manifestations associated with PCOS that have long-term consequences. Issues like depression, anxiety, reduced quality of life, and the impact of PCOS on pregnancy and post-menopause came into focus. These problems tend to be more prevalent, especially among older women with PCOS. Our study revealed a glaring lack of this knowledge, particularly among Non-RMGE (Mean 14.0 correct named symptoms, p < 0.001, multivariate analysis: B = 4.530. CI 1.379–7.680; p = 0.006). This deficiency may have profound consequences, as PCOS patients, especially during pregnancy, and in later stages of life, are often cared for in hospitals by Non-RMGE.

### 4.1 Strengths and limitations

A clear limitation of our research is its design, as it was conducted as an online survey utilizing invitation emails. In surveys, there's a potential for bias if individuals who didn't respond differ notably from those who did in terms of demographic and practice characteristics [33]. However, the issue of non-response bias might be less worrisome in surveys involving physicians, as they represent a relatively uniform population in terms of expertise, education, and perspectives [34]. Prior research has demonstrated that increased response rates did not correlate with reduced response bias in this special group of participants [33].

In our study, a substantial number of participants were recruited, ensuring robust statistical power. Compared to the study carried out by Dokras et al., the percentage of participants related to the total population remained approximately the same, reflecting the demographic diversity of the general populations of both North America and Germany. However, unlike the study by Dokras et al., no physicians from other specialties, nurses, midwives, psychologists, nutritionists, or scientific researchers were included. This allows for a nuanced analysis of PCOS in an international context.

One flaw in the study's design is the non-personalized nature of the distributed questionnaire, allowing the possibility that participants could complete the questionnaire multiple times. This lack of personalization in the questionnaire could have affected the quality and integrity of the data, as repeated entries from individual participants cannot be ruled out. There is also uncertainty about whether the incorrectly marked or unanswered questions in this online survey represent a genuine lack of knowledge and whether certain answers were left unchecked in a hectic daily routine due to time constraints or laxity.

Furthermore, the questionnaire was exclusively sent to hospitals and fertility centers, and not to outpatient practices. However, it is likely that the majority of PCOS patients, especially those without fertility issues, are treated in the outpatient settings. This limitation in the study population could potentially introduce biases into the results. A critical reflection on the representativeness of the sample in relation to the overall population of physicians and healthcare professionals is therefore important to appropriately assess the generalizability of the study results.

This study highlights a significant need for improvement in the training, especially for physicians working in hospitals, regarding PCOS. The majority of respondents expressed a preference for the implementation of a website for health professionals containing information and training on PCOS. This would represent an easily achievable measure with low-threshold access. Conducting further studies to assess whether such a measure achieves the necessary success would be advisable.

## 5 Conclusion

This study is the first to identify knowledge gaps about PCOS among physicians in Germany. The findings also highlighted the potential disparities in PCOS knowledge between hospital and fertility center settings, emphasizing the need for improved training to ensure consistent and high-quality care for PCOS patients. The participants preferred a dedicated website for health professionals, indicating a demand for easily accessible information and training resources.

## CRediT authorship contribution statement

**Konstantin Hofmann:** Writing – review & editing, Writing – original draft, Visualization, Validation, Software, Resources, Project administration, Methodology, Investigation, Formal analysis, Data curation, Conceptualization. **Melody Oehler:** Data curation. **Christian Ruckes:** Writing – review & editing, Validation, Formal analysis. **Anna Dionysopoulou:** Writing – review & editing. **Kathrin Stewen:** Writing – review & editing. **Lina Judit Schiestl:** Writing – review & editing. **Yaman Degirmenci:** Writing – review & editing. **Susanne Theis:** Writing – review & editing. **Christine Skala:** Writing – review & editing. **Annette Hasenburg:** Writing – review & editing. **Roxana Schwab:** Writing – review & editing, Visualization, Validation, Project administration, Methodology, Conceptualization.

## Ethics approval

This study was reviewed and deemed exempt from ethics approval by the committee of Rhineland Palatinate with the reference number: 2023–17215, dated September 20, 2023.

## Consent

All participants were informed that consent to participate in the study and publish their data would be assumed on completion and submission of the survey. Furthermore, the participants had to agree to participation and publication at the beginning of the survey.

## Data availability statement

The datasets generated and analyzed are available from the corresponding author on reasonable request. Due to privacy and ethical concerns, the data are not publicly available. However, de-identified data supporting the findings of this study can be made available to qualified researchers upon request, subject to approval by the Institutional Review Board.

## Declaration of generative AI and AI-assisted technologies in the writing process

During the preparation of this work, the authors used ChatGPT 3.5 (OpenAI; USA) in order to increase readability. After using this tool, the authors reviewed and edited the content as needed and take full responsibility for the content of the publication.

## Funding

This research did not receive any specific funding.

## Declaration of competing interest

The authors declare the following financial interests/personal relationships which may be considered as potential competing interests: Roxana Schwab received honoraria from Roche Pharma AG, AstraZeneca, MSD Sharp&Dohme GmbH, Sanofi GmBH and Streamedup!GmbH. If there are other authors, they declare that they have no known competing financial interests or personal relationships that could have appeared to influence the work reported in this paper.
