# Supplementary material for: Gaps in knowledge regarding the diagnostic criteria and management of PCOS in Germany: An anonymous web-based survey
Source: Heliyon. 2024 Nov 19;10(22):e40431. doi: 10.1016/j.heliyon.2024.e40431 (PMC11617859; doi:10.1016/j.heliyon.2024.e40431)
Supplement: Multimedia component 1 [file mmc1.docx]

**Supplementary material**

Supplementary material 1: Which of the following symptoms do you believe are associated with PCOS? The participants were able to select as many answer options as they wished. The percentage of given selected answers of all participants is shown.

|  | Overall |  |
| --- | --- | --- |
| Irregular menstrual cycles/periods  Reduced fertility  High blood androgen levels  Excess hair growth  Increased tendency for weight gain  Acne  Scalp hair loss/male pattern-baldness  Insulin resistance  Infertility  Hormone imbalance  Increased risk of Gestational Diabetes  Difficulty losing weight  Improvement of symptoms with exercise  Improvement of symptoms after weight loss  Endometrial cancer  Pregnancy complications  Increased risk of Cardiovascular Disease risk factors  Reduced quality of life  Depression  Improvement of symptoms with a low glycemic index (GI) diet  Cysts in ovaries  Anxiety  Sleep apnea and snoring  Body image dissatisfaction  Surgery on ovaries  Increased risk of Type 1 Diabetes  Premenstrual syndrome (PMS)  Ovarian cancer  Migraines | 198  178  169  169  151  150  149  140  139  137  132  129  127  126  125  124  113  112  94  93  84  71  68  61  59  39  34  32  26 | 96.1%  86.4%  82.0%  82.0%  73.3%  72.8%  72.3%  68.0%  67.5%  66.5%  64.1%  62.6%  61.7%  61.2%  60.7%  60.2%  54.9%  54.4%  45.6%  45.1%  40.8%  34.5%  33.0%  29.6%  28.6%  18.9%  16.5%  15.5%  12.6% |
